# Electroacupuncture versus solifenacin for women with urgency-predominant mixed urinary incontinence: a protocol for a three-armed non-inferiority randomized controlled trial

**DOI:** 10.1186/s12906-019-2784-1

**Published:** 2020-01-23

**Authors:** Yuanjie Sun, Yan Liu, Tongsheng Su, Jianhua Sun, Ying Wu, Zhishun Liu

**Affiliations:** 1 Guang’anmen Hospital, China Academy of Chinese Medical Sciences, Beijing, China; 2 Key Laboratory of Chinese Internal Medicine of Ministry of Education, Dongzhimen Hospital, Beijing University of Chinese Medicine, Beijing, China; 3 Shanxi Province Hospital of Traditional Chinese Medicine, Xi’an, China; 4 Jiangsu Province Hospital of Traditional Chinese Medicine, Nanjing, China; 5 School of Public Health, Southern Medical University, Guangzhou, China

**Keywords:** Acupuncture, Antimuscarinic drugs, Urge, Stress

## Abstract

**Background:**

Among women suffering from urinary incontinence (UI), about one-third are diagnosed with mixed urinary incontinence (MUI), among which urgency-predominant MUI causes more shame and inconvenience to patients. The treatments for urgency-predominant MUI have limited guidelines and previous studies have indicated that electroacupuncture (EA) might be a safe and effective option. The present study aims to evaluate the effect of EA on women with urgency-predominant MUI.

**Methods:**

The study is a multicentered, three-armed, non-inferiority randomized clinical trial. A total of 282 female patients with urgency-predominant MUI will be randomly divided into three groups, namely the EA group, sham electroacupuncture (SA) group, and solifenacin treatment group at a ratio of 1:1:1. Thirty-six sessions of acupuncture treatment over 12 weeks and solifenacin treatment over 36 weeks will be provided. The primary outcome will be the decrease of urgency urinary incontinence (UUI) episodes after 12-week treatment. Secondary outcomes will include changes in incontinence episodes, urinary frequency, urgency, severity of symptoms, and influence on quality of life, assessed using the International Consultation on Incontinence Questionnaire Short Form (ICIQ SF) and Overactive Bladder Questionnaire Short Form (OAB-q SF). All patients will be continuously followed up until week 36 and their allocations will be statistically analyzed.

**Discussion:**

Though placebo of solifenacin is rather difficult to access and all patients in the trial cannot be fully blinded, the present study will serve as an introduction of three-armed, randomized, non-inferiority, and sham acupuncture-controlled clinical trials to the acupuncture field, in an attempt to compare the effects of EA and solifenacin for treating women with urgency-predominant MUI.

**Trial registration:**

ClinicalTrials.gov: NCT03787654. Registered on 25 December, 2018.

## Background

Mixed urinary incontinence (MUI) is defined as a complaint of involuntary loss of urine associated with urgency, effort, or physical exertion or upon sneezing or coughing by the current International Continence Society guidelines [1]. The percentage of women suffering from the condition ranges from 14% to 61% [2]. It is regarded as urgency-predominant when a sudden sensation of voiding accompanied by uncontrolled incontinence is the dominant symptom [3]. Among women suffering from urinary incontinence (UI), about one-third have MUI with symptoms of both stress urinary incontinence (SUI) and urgency urinary incontinence (UUI) [4]. Urgency symptoms are more difficult to alter through lifestyle modifications or by controlling bladder activity than both stress-predominant MUI and balanced MUI symptoms [5, 6], resulting in more shame and inconvenience for women with urgency-predominant MUI. To relieve the symptoms, conservative treatments involving lifestyle interventions and behavioral and physical therapies are recommended by the European Association of Urology (EAU) guidelines, such as bladder training, electrical stimulation, and posterior tibial nerve stimulation. However, conservative treatments are often used as part of the treatment package because of uncertain effectiveness [4]. Other therapies for UI include surgical and pharmacological management. For urgency-predominant MUI, the success rate of surgery is 52%, 26% less than that for stress-predominant MUI [7]. Additionally, associated complications, adverse events and high costs also need to be considered when surgery is recommended. Solifenacin, the first-line medicine for urgency-predominant MUI, can reduce incontinence episodes in 24 h [8]. However, it can also induce common side effects of dry mouth, blurred vision, dry eyes, and constipation [4], and has a high medication discontinuation rate [9]. Consequently, alternative therapies with more effectiveness and safety urgently need to be explored.

Acupuncture might be a promising candidate for the treatment of urgency-predominant MUI. As previous research has indicated, electroacupuncture (EA) might be noninferior to pelvic floor muscle training (PFMT) plus solifenacin in reducing urgency incontinence episodes for women with MUI [10]. However, the trial included patients with stress-predominant MUI, urgency-predominant MUI, and balanced MUI rather than focusing on urgency-predominant MUI exclusively; hence, it is difficult to confirm whether EA is effective for the treatment of urgency-predominant MUI. In addition, the efficacy of EA cannot be clearly illustrated because of the lack of a sham acupuncture-controlled group. For this reason, the present study is a three-armed sham-controlled randomized trial aimed at evaluating the effects of EA and comparing the effects of EA and solifenacin in reducing urgency incontinence episodes for women with urgency-predominant MUI.

## Methods and analysis

### Study design

The study will be a multicentered, randomized, three-armed, non-inferiority clinical trial with sham electroacupuncture (SA) and solifenacin as controls. The null hypothesis is that the improvement (difference in the number of UUI episodes between baseline and 12-week assessment) in the EA group would be 50% or less of the difference in improvement between the solifenacin and SA groups; that is, noninferiority would be established if EA, compared with solifenacin, retains at least 50% of the efficacy of solifenacin over SA. The outcome assessors and statisticians will be blinded. Patients in the EA and SA groups will be blinded, while those in the solifenacin group will not be blinded. The study will be carried out after the ethics is approved by the Institutional Review Board. All the patients in the study will be informed about the details of the study and written informed consent will be obtained before enrollment.

In the written informed consent, the participants will be informed that if they are eligible and willing to participate in the study, they will be randomized into the EA group, SA group, or solifenacin group with the same probability. The investigators plan to enroll 282 participants, with 94 in each group. Solifenacin is an effective treatment for the UUI component, as recommended by the current guidelines, and our previous studies indicated that EA and SA are both effective for urgency-predominant MUI to some degree. In the EA group, participants will receive traditional EA at traditional acupoints, while participants in the SA group will receive shallow and minimal EA at non-acupoints.

The study is designed and reported in accordance with the Consolidated Standards of Reporting Trials (CONSORT) guidelines, the Standard Protocol Items: Recommendations for Interventional Trials (SPIRIT) Checklist, and Standards for Reporting Interventions in Controlled Trials of Acupuncture (STRICTA) recommendations. The protocol has been reviewed, revised, and supported by experts in statistics, urology, and acupuncture.

### Subjects

#### Sample size calculation

The sample size was calculated based on our previous study [10]. The expected drop-out rate is 10% and the noninferiority margin is 50% of the comparative efficacy between SA and Solifenacin. The noninferiority margin was set based on our hypothesis that EA will be associated with at least 50% of the difference in the efficacy of solifenacin as compared with that of SA.

#### Patient recruitment

This study will be conducted in six hospitals over China, specifically Guang’anmen Hospital, China Academy of Chinese Medical Sciences; Jiangsu Province Hospital of Traditional Chinese Medicine; Shanxi Province Hospital of Traditional Chinese Medicine; Guangdong Province Hospital of Traditional Chinese Medical; the First Hospital of Hunan University of Chinese Medicine; and Hengyang Hospital Affiliated to Hunan University of Chinese Medicine. Of the six hospitals, one plans to recruit 42 patients, while the other five plan to recruit 48 patients each. Patients will be recruited via newspapers, posters, and networking both from the surrounding communities and hospitals. All potentially eligible women with incontinence will be invited to participate in the trial, proceeding with a series of history taking, physical examination, specialist diagnosis, questionnaire, voiding diary, and laboratory examinations to screen and enroll the patients, as recommended by EAU guideline [4]. Eligible patients will be randomly placed in the EA, SA, or solifenacin treatment group.

Demographic information about age, marital status, menstrual status, education background, occupation, and medical history will be cautiously recorded at the baseline period. The International Consultation on Incontinence Questionnaire Short Form (ICIQ SF) and the Overactive bladder questionnaire short form (OAB-q SF) will also be used at the baseline period to assess the severity of incontinence symptoms and the influence on quality of life (QoL).

**Fig. 1:**
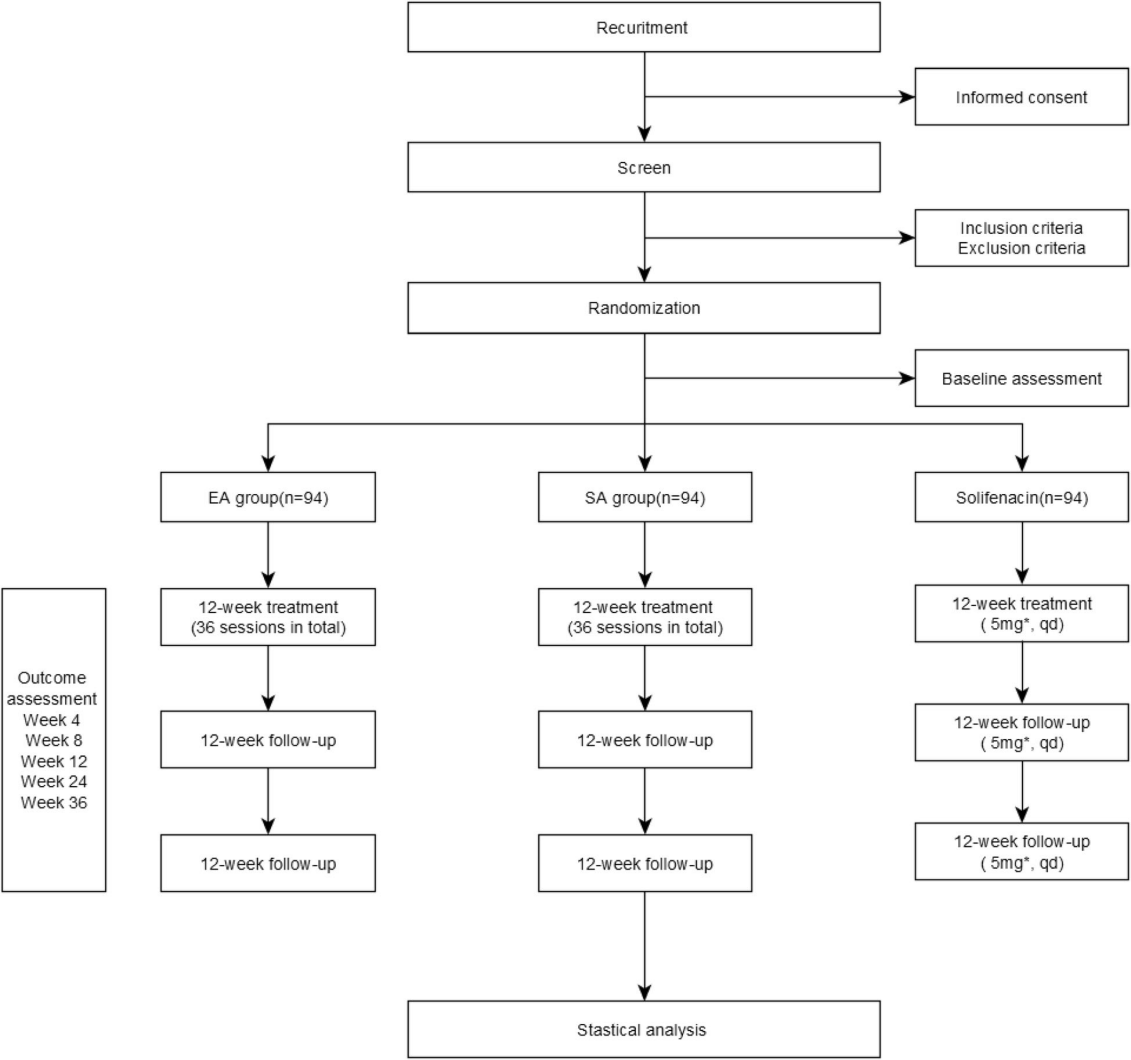
Study Flowchart. *The dose of solifenacin can be increased to 10 mg per day and can be discontinued following a comprehensive consideration of the state of incontinence symptoms and the side effects

In the EA and SA groups, patients will receive 12-week treatment and 24-week follow-up, while those in the solifenacin treatment group will take the medicine for a succession of 36 weeks. Assessments will be carried out at the baseline, at 4, 8, and 12 weeks from the baseline in the treatment period, and at 24 and 36 weeks from the baseline in the follow-up period (Fig. 1 Study Flowchart; Table 1 Outcome Assessment Schedule).

**Table 1:** Outcome Assessment Schedule

The assessment of timepoint						
Timepoint	Week 0	Week 4	Week 8	Week 12	Week 24	Week 36
Incontinence episodes in 72 h	X	X	X	X	X	X
Urgency incontinence episodes in 72 h	X	X	X	X	X	X
Stress incontinence episodes in 72 h	X	X	X	X	X	X
Other incontinence episode in 72 h	X	X	X	X	X	X
Urinary frequency in 72 h	X	X	X	X	X	X
Urinate episodes regarded as Grade 2 by PPIUS* in 72 h	X	X	X	X	X	X
Urinate episodes regarded as Grade 3 by PPIUS in 72 h	X	X	X	X	X	X
Nocturia episode in 72 h	X	X	X	X	X	X
Water input in 72 h	X	X	X	X	X	X
Pad consumption in 72 h	X	X	X	X	X	X
ICIQ SF* questionnaire	X	X	X	X	X	X
OAB-q SF* questionnaire	X	X	X	X	X	X
PGI-I* score				X		X
PGSC*		X	X	X	X	X
Expectance evaluation	X					
Blind effect evaluation				X		
Dose of solifenacin taken		X	X	X	X	X
Safety evaluation of acupuncture	X					
Adverse effect of Solifenacin	X					
Other adverse events	X					
Combination of medication	X					
Adherence evaluation	X					
Completeness of research	X					

Abbreviations: PPIUS: Patient perception of Intensity of Urgency Scale; ICIQ SF: International Consultation on Incontinence Questionnaire Short Form; OAB-q SF: Overactive bladder questionnaire short form; PGI-I: Patient global impression improvement; PGSC: Patient Global Symptom Control

#### Inclusion criteria

1. Female patients diagnosed with MUI in accordance with EAU guidelines, determined by history taking, physical examination, and specialist diagnosis [11];
2. Age between 18 and 80 years old;
3. Urge index greater than stress index, determined with the Medical, Epidemiologic, and Social aspects of Aging (MESA) questionnaire [12];
4. At least four episodes of UUI in a 3-day voiding diary;
5. UUI episodes dominate more than 50% of the total incontinence episodes in the 3-day voiding diary;
6. Positive cough stress test;
7. A voluntarily-signed written informed content.

#### Exclusion criteria

1. Having pure SUI, pure UUI, overflow UI, or neurogenic bladder;
2. Uncontrolled urinary tract infection;
3. Tumor in urinary system or pelvic organs;
4. Pelvic organ prolapse ≥ degree II;
5. Residual urine volume ≥ 100 ml;
6. Maximum flow rate<15 ml/s;
7. Receiving treatment of acupuncture or positive medications targeted at incontinence, such as antimuscarinic drugs, in the past 1 month;
8. Underwent anti-incontinence or pelvic organ surgery, including metrectomy;
9. Complications of severe diabetes or hypertension;
10. Complications of diseases in the nervous system that could hamper hypourethral function, such as multiple sclerosis, senile dementia, Parkinson’s disease, spinal cord injury, cauda equina nerve injury, and multiple system atrophy;
11. Severe complications in the cardiac, lung, cerebrum, hepatic, or renal system, psychonosology and coagulation function, or obvious cognitive disability;
12. Installation of a cardiac pacemaker;
13. Allergy to solifenacin or contraindications to antimuscarinic drug, including urinary retention, gastrointestinal peristalsis paralysis, myasthenia gravis, ulcerative colitis, and angle-closure glaucoma;
14. Allergy to metal or intolerance to EA stimulation;
15. Pregnant or planning to conceive in the next 1 year or delivered within the past 1 year.

#### Randomization

Randomization will be performed by an independent third party, Linkermed Technology Co. Ltd. (Beijing, China). Randomization sequence will be generated by computers in varying block sizes and stratified by centers, and central randomization system will be adopted to conduct the process. Once the patient is confirmed eligible to be enrolled, the study coordinator in charge of the randomization will log into the central randomization system to obtain the allocation information. Patients will be randomly allocated into the EA group, SA group, or solifenacin treatment group at a ratio of 1:1:1.

#### Blinding

Patients will be informed that they might be allocated to one of the three groups before enrollment. Patients in the EA and SA groups will be unaware of the allocation while those in the solifenacin group will not be blinded. The procedures for SA will be identical to those for EA, except that needles will be inserted into non-acupoints shallowly, with no manipulation, no sensation of *deqi,* and a minimal electric current. Patients will be treated separately to prevent communication. After 12-week treatment, the blinding effect will be evaluated.

Except for the acupuncturists, the principal investigators, co-investigators, research assistants, outcome assessors, and statisticians will all be blinded to the allocation.

#### Quality assurance of acupuncture treatment

EA and SA will be performed by acupuncturists licensed to practice traditional Chinese medicine with at least 5 years of clinical experience. Before the trial, all acupuncturists will be trained by the principle investigator (Doctor Zhishun Liu) on all details of the acupoints, insertion depth, and manipulation methods.

#### Interventions

In the EA group, the acupoints of the bilateral Zhong Liao (BL33), Hui Yang (BL35), and San Yinjiao (SP6) will be stimulated using needles with sizes of 0.30 × 75 mm and 0.30 × 40 mm, whereas in the SA group, sham Zhong Liao (BL33), Huiyang (BL35), and San Yinjiao (SP6) will be stimulated using needles with sizes of 0.30 × 40 mm (Details in Table 2).

**Table 2:** Details of interventions

		EA Group	SA Group
Retention time		30 min	
Treatment sessions		36 sessions	
Frequency and duration of treatment		3 sessions per week (every other day ideally) for 12 week	
Type of tools		SDZ-V electronic apparatus and Huatuo Brand disposable sterilized needles	
The first Pair of acupoints	Names	Zhong Liao (BL33)	Sham Zhong Liao (BL33)
	Location	Third foramina sacralia posteriora	1cun (≈10 mm) horizontally outwardly lateral to Zhong Liao (BL33)
	Needle type	0.30 × 75 mm	0.30 × 40 mm
	Depth of insertion	60~70 mm	2-3 mm to stand still
	Response sought	De qi	No sensation of de qi
	Manipulating method	The needle will be penetrated into skin at the area 0.5-1 cm upside and outside away from Zhong Liao (BL33), inserted further at a degree of 45° inward and downward into the foramina and lifted, thrusted and thrilled gently and evenly for three times.	Without any manipulation
	Electric parameters	Continuous wave, 20 Hz, 2 mA–6.5 mA. Adjust from zero to the degree which patients can tolerate.	Continuous wave, 20 Hz, 0.1–0.3 mA.
The second pairof acupoints	Names	Hui Yang (BL35)	Sham Hui Yang (BL35)
	Location	0.5 cun (≈10 mm) lateral to coccyx end	1cun (≈10 mm) horizontally outwardly lateral to Hui Yang (BL35)
	Needle type	0.30 × 75 mm	0.30 × 40 mm
	Depth of insertion	60~70 mm	2-3 mm to stand still
	Response sought	De qi	No sensation of deqi
	Manipulating method	The needle will be penerated into the skin at the area right beside the coccyx end, inserted further outward and upward slightly and lifted, thrusted and thrilled gently and evenly for three times.	Without any manipulation
	Electric parameters	Continuous wave, 20 Hz, 2 mA–6.5 mA. Adjust from zero to the degree which patients can tolerate	Continuous wave, 20 Hz, 0.1–0.3 mA.
The third pair of acupoints	Names	San Yinjiao (SP6)	Sham San Yinjiao (SP6)
	Location	Posterior border of medial tibia, 3cun above medial malleolus tip	0.5cun (≈10 mm) horizontally behind San Yinjiao (SP6)
	Needle type	0.30 × 40 mm	0.30 × 40 mm
	Depth of insertion	25-30 mm	2-3 mm to stand still
	Response sought	De qi. Electricity-shock feelings should be avoided.	No sensation of deqi
	Manipulating method	The needle will be penetrated downward and lifted, thrusted and thrilled gently and evenly for three times.	No manipulation
	Electric parameters	Continuous wave, 20 Hz, 1–3.5 mA. Adjust from zero to the degree which patients can tolerate	Continuous wave, 20 Hz, 0.1–0.3 mA

#### Solifenacin treatment group

In the solifenacin treatment group, patients will take 5 mg solifenacin orally per day for a succession of 36 weeks.

The five-degree Patient Global Symptom Control (PGSC) [13] will be applied at weeks 4, 8, 12, 24, and 36 to assess whether urinary leakage is adequately controlled by solifenacin, with a score of 1 indicating strong disagreement and a score of 5 indicating strong agreement. Patients can continue with the current dose of solifenacin if they score 4 or 5, and the dose can be increased to 10 mg per day if they score 1–3 points. However, the final decision will be made by the doctors under comprehensive consideration of the PGSC score and side effects. Solifenacin can be discontinued at any time in the event of severe side effects. The dose changes, discontinuation, and side effects will be recorded clearly in the case report form (CRF).

#### Concomitant treatment

To avoid other influences on the function of the lower urinary tract and incontinence symptoms, patients will be recommended not to receive any other treatments. If used, the treatment or medication should be clearly recorded in the CRF.

### Measures

#### Outcome assessment

##### Primary outcome measure

The primary outcome is the change in UUI episodes during an average 24-h period at 12 weeks from the baseline (after the first treatment), recorded in a 3-day voiding dairy.

##### Secondary outcomes

UUI episodes, urinary frequency, urgency, nocturia, pad consumption, and volume drunk in an average 24-h period will be recorded in a 3-day voiding diary both during the treatment period (week 4, week 8, week 12) and the follow-up period (week 24, week 36). The 3-day voiding diary is a reliable, objective, and sensitive measurement recommended by EAU guidelines to monitor changes in incontinence. Urination, urgency, incontinence, drinking, and pads consumed are recorded. Urgency will be evaluated using the Patient Perception of Intensity of Urgency Scale (PPIUS), which is recommended by the European Medicines Agency [14]. Voiding at levels of 3 and 4 are regarded as urgency episodes [15], while levels 1 and 2 are regarded as normal or a strong desire to void [14].

In addition, the ICIQ SF and OAB-q SF scores will be applied as outcome measurements both during the treatment period (week 4, week 8, week 12) and follow-up period (week 24, week 36). The ICIQ SF is a questionnaire developed by the International Consultation on Incontinence [16] to evaluate the severity of incontinence and impact on QoL in the past 4 weeks. It contains four items: frequency, amount of leakage, impact of UI on QoL, and a separate item to indicate the cause of incontinence. The score is the sum of the first three items and the total score ranges from 0 to 21, with a higher score indicating worse symptoms and greater impact on QoL. A Chinese version has been translated and validated [17]. OAB-q SF is a validated questionnaire used to assess the burden of OAB symptoms and the effect on QoL in the past 4 weeks. The questionnaire includes items on coping, sleep, and emotional interactions [18]. The scores are transformed to a 0- to 100-point scale, with higher symptom-severity scores indicating worse symptoms and higher QoL scores indicating a better QoL. A Chinese version of OAB-q SF has also been validated [19].

Patient global impression improvement (PGI-I) will be applied in week 12 and week 36 to measure the effects of therapy subjectively. PGI-I is a scale ranging from 1 to 7, with 1 indicating very much better, 2 indicating much better up to 7 indicating very much worse [20].

Residual urinary volume will be retested with abdominal B-ultrasound in week 12 from the baseline.

The expectations of patients regarding acupuncture and Solifenacin will be assessed before the first treatment, and whether the patients in the EA and SA groups are blinded successfully will be assessed within 5 min after any treatment in the last week. Participants will be told that they may receive one of two treatments, traditional EA treatment, where traditional acupoints are inserted relatively deeply, or minimal EA treatment, where non-acupoints are inserted shallowly and gently. Then they will be asked to guess if they received traditional EA, and choose from answers of yes or no.

#### Safety assessment

##### Side effects associated with acupuncture

Unintended events and feelings related to EA and SA will be recorded in the CRF, including broken needles, fainting, bleeding, bruising, infection and aposteme, unbearable ache (VAS ≥ 8), vomiting, nausea, palpitations, dizziness, headache, anorexia, and insomnia.

##### Side effects associated with solifenacin

The side effects induced by Solifenacin will be recorded in the CRF, including dry mouth, dry eyes, and constipation.

##### Adverse events

Except from the side effects induced by EA, SA, and solifenacin, all other adverse events will be recorded in the CRF throughout the trial, and their relationships with the intervention and controls will be assessed.

#### Adherence assessment

The sessions of treatments will be recorded in the CRF to assess the adherence of patients.

### Data management

The CRF will be filled in before the input of data into the Electronic Data Capture (EDC) System, which will be developed by a third independent corporation (Linkermed Technology Co. Ltd). The database will be protected by a password and only the principal investigator will have access to the final dataset.

The EDC system will be locked once the study is completed. The CRF and dataset will be preserved for 5 years. Data from the trial will be shared upon request to the corresponding author anonymously, excluding the private information of patients.

### Statistical analysis

For the primary hypothesis, we will compare the decrease in UUI episodes among the three groups. A decrease in number will indicate less incontinence. Additionally, we will use a modified *t*-test based on the retention of effect method to analyze whether EA is noninferior to solifenacin [21]. In detail, to establish the assay sensitivity of the trial, a *t*-test investigating the superiority of solifenacin over SA will be performed first by comparing the decrease in the number of UUI episodes in the solifenacin and SA groups. After superiority has been established, a modified *t*-test will be used to assess whether the difference in the numbers between the EA and SA groups is more than 50% of the mean difference between the numbers in the solifenacin and SA groups. An appropriate Fieller’s confidence interval can also be provided [21]. If the *P* Value is equal to or less than 0.025 or if the lower boundary of the confidence interval around the mean difference between the numbers in the EA group versus that in the solifenacin group exceeds this value, then noninferiority will be established. After the noninferiority assessment, traditional *t*-tests will be used for superiority analyses, including a decrease in the number of UUI episodes for solifenacin versus EA, and EA versus SA.

A mixed-effect model or Wilcoxon rank-sum test will be used to assess the longitudinal continuous variables. A generalized linear model or logistic regression will be performed to assess the rates of response. Chi-square or Fisher’s exact tests will be used to compare the frequency of AEs between groups.

Primary outcome analysis will be performed in the intention-to-treat (ITT, randomized participants) and per-protocol datasets (PP, ITT participants who received at least one treatment session without major violation of the protocol). Missing data will be imputed using the multiple imputation method under the missing-at random assumption. Secondary and safety outcomes will be assessed in ITT populations.

Since all analyses other than the primary analysis are considered descriptive, no adjustments will be made for multiple testing, and *P* values should be interpreted accordingly. Analyses will be performed using SAS version 9.4 (SAS Institute Inc) with a two-sided *P* value of less than .05 considered significant.

## Discussion

This study aims to demonstrate that EA is noninferior to solifenacin in reducing UUI episodes in women with urgency-predominant MUI after 12-week treatment.

At present, the diagnostic criteria for urgency-predominant MUI have not been clearly defined. In this study, women will be diagnosed with MUI through history taking, physical examination, questionnaires, voiding diary, specialist diagnosis, and laboratory tests as recommended by the 2018 EAU guidelines. The MESA questionnaire and 3-day voiding diary will be applied to doubly ensure urgency-predominance, which needs to be studied further.

Voiding diaries of 3 and 7 days are reliable in monitoring changes in voiding and incontinence and are thus used as the main tools for outcome assessment. Since urgency is a bothersome symptom in urgency-predominant MUI, overactive bladder, PPIUS, and OAB-q SF are applied to evaluate the severity and the influence on QoL. In this study, both subjective and objective measurements will be applied to assess the outcomes.

In two-arm non-inferiority trials, the choice of a non-inferiority margin is usually based on differences between the active comparator and placebo from previous trials and meta-analyses [22]. However, this approach is problematic for this study because the active comparator in the trial will not necessarily have the same efficacy as previous results in the literature.

A two-arm non-inferiority trial in which a placebo arm is not included is not appropriate due to the lack of assay sensitivity.

The non-inferiority design with a three-arm trial allows indexing of the therapeutic effect of EA based on the performance of Solifenacin and SA within the trial, instead of using external references, as further discussed. Therefore, it is considered the “gold standard” of non-inferiority trials according to the US Food and Drug Administration (FDA) and the European Medical Agency (EMA) [23].

However, the placebo of Solifenacin is rather difficult to access in China, making the blinding of all patients impossible in a three-armed non-inferiority trial. Therefore, the efficacy of solifenacin (solifenacin plus SA) compared with a placebo (Placebo plus SA) cannot be assessed.

## Trial status

The recruitment has already started.

## Supplementary information

**Additional file 1.** SPIRIT guidelines.

## Data Availability

The datasets used and/or analyzed during the current study are available from the corresponding author on reasonable request anonymously.

## Abbreviations

ICIQ SF: International Consultation on Incontinence Questionnaire Short Form
MUI: Mixed urinary incontinence
OAB-q SF: Overactive bladder questionnaire short form
PGI-I: Patient global impression improvement
PGSC: Patient Global Symptom Control
PPIUS: Patient Perception of Intensity of Urgency Scale
SUI: Stress urinary incontinence
UI: Urinary incontinence
UUI: Urgency urinary incontinence
